# Beyond Acquisition: Quantifying the Annual Carrying Cost of Long-Term Biospecimen Storage

**DOI:** 10.3390/biotech15040081

**Published:** 2026-09-23

**Authors:** T. T. Olson, P. J. Catterson, K. K. Mohr, C. S. Dulny, M. V. Olson

**Affiliations:** Johns Hopkins BioBank, Johns Hopkins Integrated Genomic Center, The Johns Hopkins University School of Medicine, 600 N. Wolfe Street, Baltimore, MD 21287, USA

**Keywords:** biobanking, biospecimens, long-term storage, annual carrying cost, biobank sustainability, biospecimen utilization, storage capacity, lifecycle management

## Abstract

**Background:** Long-term biospecimen preservation requires sustained institutional investment in storage equipment, energy or cryogen, monitoring, maintenance, and inventory management. Although biobank economics and biospecimen utilization have been examined independently, the recurring direct cost associated with maintaining existing inventories in relation to storage capacity and research utilization remains less well characterized. We introduce Annual Carrying Cost (ACC) as an operational metric for quantifying this continuing institutional investment. **Methods:** A retrospective observational analysis was conducted within a large academic biobank using long-term storage infrastructure and retrieval data from 2023–2025. ACC incorporated annualized equipment and monitoring-probe costs, energy and cryogen consumption, maintenance and repair, environmental monitoring, and inventory management. Storage capacity was characterized according to inventory-bearing, online contingency, and offline/staged reserve capacity. ACC was normalized to available and occupied vial positions and evaluated in relation to annual research-directed vial-equivalent retrievals. **Results:** Sixteen storage units provided 832,120 vial positions, of which 272,476 were occupied (32.7%). Eight inventory-bearing units were 56.1% occupied, while 41.6% of total capacity was intentionally maintained as online contingency or offline/staged reserve infrastructure. Estimated ACC was $131,252 annually, comprising annualized equipment and monitoring probes (40.5%), energy and cryogen (30.8%), environmental monitoring (14.4%), maintenance and repair (8.4%), and inventory management (6.0%). ACC corresponded to $0.16 per available vial position and $0.48 per occupied vial position annually. Research-directed retrieval volume varied more than 13-fold across the study period, from 217,348 vial equivalents in 2023 to 16,414 in 2025, resulting in retrieval-normalized ACC ranging from $0.60 to $8.00 per vial-equivalent retrieved. **Conclusions:** Long-term biospecimen retention creates a measurable recurring institutional obligation that persists despite variation in annual research utilization. ACC provides a practical framework for characterizing this investment and distinguishing routinely occupied storage from infrastructure maintained for resilience and future capacity. When considered alongside scientific value, utilization, uniqueness, and anticipated future demand, ACC may support more informed capacity planning, lifecycle management, resource allocation, and sustainable long-term biospecimen stewardship.

## 1. Introduction

Institutional biobanks represent a substantial and enduring investment in biomedical research infrastructure. Collectively, these repositories maintain millions of biospecimens that support basic, translational, and clinical investigation while preserving biological resources for future scientific discovery [1,2,3,4,5,6,7,8]. Over the past two decades, advances in biospecimen collection, processing, preservation, quality management, and governance have strengthened the reliability of these resources and established biobanks as essential components of the research enterprise [2,9].

The value of long-term biospecimen preservation, however, is accompanied by a continuing institutional obligation. Biospecimens maintained at ultra-low temperatures require specialized storage equipment, continuous environmental monitoring, preventive maintenance, emergency backup systems, dedicated personnel, and sustained energy or liquid nitrogen consumption [3,10,11,12,13,14,15,16]. Unlike specimen acquisition and processing, for which many costs occur at or near the time of collection, storage creates recurring resource requirements that continue for as long as the specimens are retained [3,10]. As collections accumulate over time, these recurring requirements can represent a substantial and often underrecognized component of the institutional investment in biobanking [3,10,11,17].

This challenge is amplified by the long retention periods characteristic of many biobanks. Biospecimens may remain in storage for years or decades, including periods during which they are infrequently accessed [17,18,19,20,21,22]. Long-term retention may be entirely appropriate, particularly for longitudinal studies, rare diseases, unique cohorts, or irreplaceable clinical specimens. Nevertheless, every retained specimen continues to consume finite storage capacity and requires infrastructure to preserve it, regardless of how frequently it is retrieved [11,17,18,19]. Consequently, the operational burden of a biobank is determined not only by the acquisition of new specimens but also by the cumulative responsibility associated with maintaining its existing inventory.

Despite this continuing investment, relatively little attention has been directed toward quantifying the recurring operational cost of biospecimen retention in relation to actual utilization. Previous studies have examined the costs associated with establishing and operating biobanks [3,4,23,24], yet institutional decisions regarding storage expansion, equipment replacement, and long-term capacity planning are often made without standardized measures describing the annual direct cost of maintaining existing inventories. As repositories confront aging infrastructure, increasing storage demands, sustainability priorities, and constrained research budgets, understanding this ongoing burden is becoming increasingly important [10,17,24].

In this study, we introduce Annual Carrying Cost (ACC) as an operational metric representing the estimated annual direct institutional investment required to maintain biospecimens under conditions suitable for future research use following their initial acquisition (Figure 1). ACC is intended to characterize the recurring direct cost of specimen preservation rather than the total institutional cost of biospecimen ownership or the scientific value of individual collections. When considered alongside measures of specimen retrieval, ACC provides a practical means of examining the relationship between long-term storage infrastructure, continuing institutional investment, and utilization.

We applied this framework to multiple long-term biospecimen storage platforms within a large academic biobank and compared estimated ACC with specimen retrieval activity over a three-year period. By quantifying the resources required to sustain existing biospecimen inventories and examining these costs in relation to utilization, this study aims to provide an operational framework for institutional planning, resource allocation, capacity management, and sustainable long-term biospecimen stewardship.

## 2. Materials and Methods

### 2.1. Study Design

A retrospective observational study was conducted to characterize the operational infrastructure and annual costs associated with long-term biospecimen storage within the Johns Hopkins Biobank. The study quantified the Annual Carrying Cost (ACC) of multiple biospecimen storage platforms and examined this investment in relation to storage capacity and specimen retrieval activity over a three-year study period (2023–2025). The objective was to estimate the annual institutional cost of sustaining long-term biospecimen storage and to characterize this investment across storage technologies with differing capital requirements, operating costs, storage capacities, and expected useful lives.

### 2.2. Biospecimen Storage Infrastructure and Capacity

The Johns Hopkins Biobank maintains biospecimens using multiple long-term storage technologies that differ in both storage temperature and cooling technology. Storage systems included vapor-phase liquid nitrogen (LN_2_) systems (Taylor Wharton 80K, Taylor Wharton 40K, Huntsville, AL, USA and MVE World 800 Series, Burnsville, MN, USA), LN_2_-cooled −80 °C storage (MVE Vario, MVE Biological Solutions, Ball Ground, GA, USA), and mechanically refrigerated −80 °C ultra-low-temperature storage (PHCbi TwinGuard upright and chest freezers, Tokyo, Japan). This configuration permitted evaluation of storage platforms with differing temperature conditions, cooling technologies, storage capacities, operating requirements, and expected useful lives.

Repository inventory was assessed at fiscal year-end (30 June) for each year of the study period, providing annual cross-sectional snapshots of available storage capacity and occupied vial positions. Because each assessment represents the repository inventory at that fiscal year-end, changes in occupancy between annual assessments reflect the net effect of ongoing specimen additions and removals.

For each storage unit, equipment characteristics included storage technology, manufacturer and model, acquisition cost, year placed into service, expected useful life, and nominal usable storage capacity. Inventory data included individually inventoried biospecimen vials and inventory boxes. For box-level inventory records, each 9 × 9 inventory box was standardized to 81 vial-equivalent positions based on nominal box capacity. This conversion was used as an operational normalization to permit consistent comparison across storage configurations and specimen formats; individual boxes varied in both actual occupancy and the type of material stored and were not assumed to contain 81 individual vials.

Storage units were classified according to operational function at each fiscal year-end inventory assessment as inventory-bearing, online contingency, or offline/staged reserve capacity. Inventory-bearing units were maintained at operating conditions and contained routine biospecimen inventory. Online contingency units were maintained at operating temperature but intentionally contained no routine inventory, providing immediately available capacity for emergency specimen transfer following equipment failure and for short-term fluctuations in storage demand. Offline/staged reserve units contained no inventory and were not maintained at operating conditions but remained installed and available for activation in response to future inventory requirements.

Total available capacity included the nominal usable capacity of all inventory-bearing, online contingency, and offline/staged reserve units. Overall capacity utilization was calculated as occupied vial positions divided by total available capacity. Occupancy of inventory-bearing storage was also calculated separately to distinguish utilization of actively populated storage systems from capacity intentionally maintained for contingency and future storage requirements.

### 2.3. Annual Carrying Cost

Annual Carrying Cost (ACC) was defined as the estimated annual institutional cost of maintaining biospecimens under conditions suitable for future research use following their initial acquisition and processing. ACC incorporated annualized storage-equipment costs and recurring infrastructure expenses directly attributable to long-term specimen preservation, monitoring, and traceability (Figure 1).

Equipment cost included the acquisition cost of each storage unit together with dedicated environmental monitoring probes acquired as part of the storage system. Equipment and probe costs were distributed across the expected useful life of the corresponding storage unit using straight-line annualization, with no residual value assumed. Annualized equipment cost was used as an operational costing measure and does not represent institutional accounting depreciation.

Annualized equipment costs were retained for inventory-bearing, online contingency, and offline/staged reserve systems because each represented infrastructure maintained as part of the repository’s long-term storage capacity. Energy and cryogen costs were attributed only to units maintained at operating conditions.

Recurring operating expenses included electricity for mechanically refrigerated −80 °C storage, LN_2_ for LN_2_-dependent storage systems, preventive maintenance and repair, environmental monitoring, and repository inventory-management infrastructure required to maintain specimen identification, location, and traceability. Annual LN_2_ expense was based on actual institutional expenditures. Electricity costs were calculated using equipment-specific consumption and institutional electricity rates, with manufacturer-reported consumption used when measured data were unavailable. Maintenance and repair expenses were based on attributable repository costs. Environmental monitoring costs represented the portion of institutional monitoring services attributable to biobank storage infrastructure.

Repository-wide ACC was calculated as:

ACC = Annualized Equipment Cost + Energy/Cryogen + Maintenance and Repair + Environmental Monitoring + Inventory Management

Personnel effort, facility and space costs, facility-level emergency and backup infrastructure, institutional overhead, indirect costs, and costs associated with specimen acquisition, processing, and initial accessioning were excluded. ACC therefore represents the annual infrastructure and operating cost directly associated with maintaining long-term biospecimen storage rather than the comprehensive institutional cost of biobank operation.

To account for differences between storage capacity and current inventory, ACC was normalized using two complementary measures:

ACC per Available Vial Position = Annual ACC/Total Available Vial Positions

ACC per Occupied Vial Position = Annual ACC/Total Occupied Vial Positions

The first measure represents the annual investment associated with maintaining the repository’s total storage infrastructure, including contingency and staged reserve capacity. The second relates the same annual investment to the capacity occupied by the existing biospecimen inventory.

All monetary values reported in the present analysis are expressed in U.S. dollars (USD). The ACC framework itself is currency-agnostic and is intended to be calculated using locally relevant institutional costs. Absolute ACC values are therefore expected to vary across institutions and geographic regions according to factors including storage technologies, energy and cryogen costs, equipment and maintenance expenses, and local operating environments. Accordingly, the framework is intended to standardize the categories of recurring investment considered rather than establish a universal monetary benchmark.

These normalization measures do not allocate individual ACC components to specific storage positions. Rather, they relate the total repository-level annual carrying cost to two complementary measures of storage utilization: the infrastructure maintained and the inventory occupying that infrastructure. Inventory-management costs therefore represent repository-level activities required to maintain specimen identification, location, and traceability and are not assigned directly to individual occupied or unoccupied positions.

Costs associated with specimen acquisition, processing, and initial accessioning were excluded because ACC is intended to quantify the recurring post-acquisition cost of maintaining biospecimens in long-term storage; however, subsequent additions and removals are reflected in the occupied inventory measured at each annual assessment.

### 2.4. Specimen Retrieval and Utilization

Specimen retrieval activity was assessed using biospecimen distribution records maintained by the Johns Hopkins Biobank and evaluated separately for fiscal years (FY) 2023, 2024, and 2025, with each fiscal year defined as July 1 through June 30. Retrievals represented biospecimens removed from long-term storage for research-directed purposes.

Retrieval volume was standardized as vial-equivalent retrievals to permit comparison across inventory configurations and with storage-capacity measures. Individually retrieved vials were counted directly, while retrieved inventory boxes were converted to vial equivalents using the standardized capacity of 81 vial-equivalent positions per box. Annual vial-equivalent retrieval volume was evaluated separately for each fiscal year to characterize year-to-year variation in research-directed retrieval activity.

ACC was evaluated in relation to annual retrieval activity as:

ACC per Vial-Equivalent Retrieved = Annual ACC/Annual Vial-Equivalent Retrievals

Retrieval-normalized ACC represents the annual carrying cost relative to the volume of biospecimen material retrieved for research during a given year. It provides an index of the relationship between the relatively persistent annual cost of maintaining long-term storage infrastructure and the level of research-directed utilization occurring during that year. It should not be interpreted as the marginal or operational cost of performing an individual specimen retrieval.

### 2.5. Study Assumptions

Expected useful life was obtained from manufacturer specifications and institutional equipment-replacement planning documentation where available. Storage units classified as online were assumed to operate continuously during the assessment period. Actual institutional expenditures or measured consumption were preferentially used for recurring operating costs; manufacturer-reported consumption was used when directly measured data were unavailable.

Offline/staged reserve units were assumed to incur annualized equipment costs but no energy or cryogen expense while not maintained at operating conditions. Online contingency systems incurred both annualized equipment and applicable operating costs because they were maintained continuously at operating temperature as part of repository emergency preparedness.

Only costs that could be reasonably attributed to the evaluated biobank storage infrastructure were included. Costs shared with equipment or activities outside the biobank were apportioned where an attributable biobank component could be established.

### 2.6. Data Analysis

Storage units served as the primary unit of analysis for characterization of infrastructure, capacity, and operational status, with results subsequently summarized across the repository and, where appropriate, by storage technology. Descriptive statistics were used to characterize storage capacity, biospecimen inventory, occupancy, annualized equipment cost, recurring operating expenses, total ACC, storage-normalized ACC, and specimen retrieval activity.

Retrieval activity was evaluated separately for 2023, 2024, and 2025 and across the three-year study period to characterize temporal variation in utilization. Retrieval-normalized ACC was calculated for each fiscal year and across the full study period. Results are presented as descriptive operational metrics intended to characterize relationships among long-term storage infrastructure, institutional investment, storage capacity, and specimen utilization. These measures are not intended to assess the scientific value or future research potential of individual biospecimens or collections.

## 3. Results

### 3.1. Characteristics, Capacity, and Utilization of Long-Term Biospecimen Storage Infrastructure

The evaluated long-term biospecimen storage infrastructure comprised 16 storage units with a combined nominal capacity of 832,120 vial positions across vapor-phase liquid nitrogen (LN_2_), LN_2_-cooled −80 °C, and mechanically refrigerated −80 °C storage systems (Table 1). At the time of inventory assessment, 272,476 vial-equivalent positions were occupied, including 65,035 individually inventoried vials and 2561 storage boxes, with each box standardized to 81 vial-equivalent positions. Overall physical capacity utilization across the storage infrastructure was 32.7%.

Storage capacity reflected multiple operational functions rather than routine inventory storage alone. Of the total nominal capacity, 485,720 vial positions (58.4%) were located within eight inventory-bearing storage units, of which 272,476 positions were occupied, corresponding to 56.1% occupancy. An additional 116,800 positions (14.0%) were maintained online at operating temperature as contingency capacity but intentionally contained no routine inventory. These systems provided immediately available capacity for emergency specimen transfer following equipment failure and for short-term fluctuations in storage demand. The remaining 229,600 positions (27.6%) were maintained offline as staged reserve capacity available for activation in response to future inventory requirements.

Collectively, online contingency and offline/staged reserve systems represented 346,400 vial positions, or 41.6% of total physical storage capacity. Thus, although 32.7% of total repository capacity was occupied at the time of assessment, a substantial proportion of apparently unoccupied capacity represented intentionally maintained infrastructure supporting emergency response, operational resilience, and changes in storage demand rather than routinely available unused storage.

### 3.2. Annual Carrying Cost of Long-Term Biospecimen Storage

The estimated Annual Carrying Cost (ACC) of the evaluated long-term biospecimen storage infrastructure was $131,252 per year. Annualized equipment costs, including dedicated environmental monitoring probes, represented the largest component of ACC at $53,100 (40.5%), followed by energy and cryogen costs of $40,381 (30.8%). Together, these components accounted for approximately 71% of total ACC (Figure 2).

The remaining approximately 29% of ACC reflected operational systems required to protect, monitor, maintain, and manage the stored inventory. Environmental monitoring accounted for $18,924 (14.4%), maintenance and repair for $10,981 (8.4%), and inventory management for $7865 (6.0%) annually.

### 3.3. Storage-Normalized Annual Carrying Cost

When ACC was normalized to the repository’s total nominal capacity of 832,120 vial positions, the annual carrying cost was approximately $0.16 per available vial position. When normalized to the 272,476 vial-equivalent positions occupied by the existing biospecimen inventory, ACC was approximately $0.48 per occupied vial position per year.

The difference between these measures reflects the inclusion of infrastructure maintained for purposes beyond routine inventory storage. Of the total nominal capacity, 346,400 vial positions (41.6%) were maintained as online contingency or offline/staged reserve capacity. Thus, normalization to total available capacity distributes ACC across the full physical infrastructure being maintained, whereas normalization to occupied capacity relates the same annual investment to the biospecimen inventory currently stored.

### 3.4. Biospecimen Retrieval and Utilization

Biospecimen retrieval volume varied substantially across the three-year study period. A total of 333,066 vial-equivalent retrievals were recorded from 2023 through 2025, including 217,348 in 2023, 99,304 in 2024, and 16,414 in 2025. Annual retrieval volume therefore varied more than 13-fold across the study period, reflecting substantial year-to-year variation in research-directed retrieval activity (Figure 3).

When annual ACC was evaluated relative to retrieval volume, retrieval-normalized ACC was approximately $0.60 per vial-equivalent retrieved in 2023, $1.32 in 2024, and $8.00 in 2025. Thus, the increase in retrieval-normalized ACC across the study period reflects declining annual retrieval volume rather than an increase in the underlying annual carrying cost. Across the full three-year period, the cumulative carrying cost was $393,756, corresponding to approximately $1.18 per vial-equivalent retrieved. This variation in retrieval-normalized ACC occurred despite the continuing requirement to maintain the underlying storage infrastructure throughout the study period.

## 4. Discussion

Long-term biospecimen preservation represents a continuing institutional commitment that extends well beyond the initial acquisition and processing of specimens. In this study, we introduce Annual Carrying Cost (ACC) as an operational metric for quantifying the recurring direct institutional investment required to maintain biospecimens under conditions suitable for future research use. Across the evaluated storage infrastructure, ACC was estimated at $131,252 annually and included annualized equipment costs, energy or cryogen consumption, environmental monitoring, maintenance and repair, and inventory management. Importantly, this carrying obligation persisted despite substantial year-to-year variation in specimen retrieval. These findings demonstrate that long-term biospecimen retention requires sustained infrastructure investment that is not directly proportional to research utilization within any individual year.

Previous approaches to biobank economics have appropriately focused on sustainability, cost recovery, business planning, and comprehensive operating costs (3, 5–9, 15), while studies of biospecimen utilization have examined patterns of access and the challenges associated with defining and measuring use (10–14). ACC is intended to complement rather than replace these approaches. Its distinguishing feature is the explicit characterization of the recurring post-acquisition investment required to sustain existing biospecimen inventories and the normalization of that investment to both storage capacity and research-directed retrieval activity. This provides a framework for examining three related but distinct dimensions of mature biobank operations: the infrastructure maintained, the inventory occupying that infrastructure, and the extent to which stored material is accessed for research. By separating the continuing obligation of retention from the broader costs of operating a biobank, ACC provides an operational lens through which institutions can evaluate long-term storage infrastructure and utilization together.

This distinction may be particularly relevant as biobanks mature. Repositories established over many years may accumulate large inventories assembled through individual research studies, prospective collection programs, clinical trials, disease-specific initiatives, and institutional collection efforts. Although the scientific rationale for acquisition may be well defined at the time of collection, future utilization cannot always be predicted. Some collections may experience frequent use, whereas others may remain largely dormant for extended periods before becoming scientifically valuable. Long periods without retrieval therefore should not, by themselves, be interpreted as evidence that specimens lack value. Rare disease collections, longitudinal cohorts, specimens associated with long-term clinical outcomes, and materials that would be difficult or impossible to replace may appropriately warrant prolonged retention despite infrequent access. Nevertheless, continued retention carries a measurable resource requirement, creating a need to consider both potential future scientific value and the infrastructure necessary to preserve that opportunity.

The distinction between physical capacity and operational function was also evident in this analysis. Although only 32.7% of the repository’s total nominal storage capacity was occupied at the time of assessment, 41.6% of total capacity was intentionally maintained as online contingency or offline/staged reserve infrastructure. Among inventory-bearing units, occupancy was 56.1%. These findings illustrate an important limitation of using physical occupancy alone as a measure of biobank efficiency. Apparently unoccupied capacity may serve essential operational functions rather than represent excess capacity. Online contingency infrastructure provides resilience following equipment failure and accommodates short-term fluctuations in storage demand, while staged reserve capacity allows repositories to respond to future requirements without immediate infrastructure expansion. For repositories maintaining unique or irreplaceable specimens, the ability to respond rapidly to storage-system failure is itself an important component of biospecimen protection. The proportion of capacity assigned to these functions will necessarily vary according to storage technology, collection characteristics, institutional resources, risk tolerance, and local operating requirements. Consequently, maximizing occupancy without accounting for contingency and reserve requirements could inadvertently reduce operational resilience.

The composition of ACC further demonstrates that the continuing investment required for biospecimen preservation extends beyond storage equipment itself. Annualized equipment costs and energy or cryogen consumption accounted for approximately 71% of ACC, while environmental monitoring, maintenance and repair, and inventory management accounted for the remaining approximately 29%. These latter functions are integral to maintaining specimens as usable research resources. Temperature-controlled storage alone is insufficient if equipment performance cannot be monitored, failures cannot be addressed, or specimens cannot be reliably located and linked to their associated inventory records. In this context, inventory management is part of the infrastructure of preservation: a specimen that remains physically intact but cannot be reliably identified or located has substantially diminished practical research utility. ACC therefore conceptualizes long-term storage not simply as maintenance of temperature, but as the combination of infrastructure and operational systems required to preserve specimen integrity, traceability, and accessibility.

Normalization of ACC to storage capacity provides complementary perspectives on this infrastructure investment. In the present study, ACC corresponded to approximately $0.16 per available vial position and $0.48 per occupied vial position annually. Neither measure should be interpreted as a universal benchmark or as the “price” of storing an individual specimen. ACC per available position distributes the recurring direct investment across the repository’s total maintained storage capacity, including infrastructure reserved for contingency and future demand, whereas ACC per occupied position relates the same investment to the inventory currently being maintained. Reporting both measures may be useful for evaluating changes within a repository over time, assessing storage expansion, and determining whether existing infrastructure can accommodate future collection requirements before additional capacity is acquired.

Relating ACC to specimen retrieval provides a third and distinct perspective. Vial-equivalent retrieval volume varied more than 13-fold during the three-year study period, from 217,348 in 2023 to 16,414 in 2025, while the underlying storage infrastructure continued to be maintained. Correspondingly, retrieval-normalized ACC ranged from approximately $0.60 to $8.00 per vial-equivalent retrieved. These values do not represent the operational cost of performing an individual retrieval. Rather, they illustrate how a relatively persistent carrying obligation is distributed across markedly different levels of annual research activity. Individual large-scale studies or collection-level distributions may substantially influence retrieval volume in a particular year, while the infrastructure required to maintain the remaining inventory persists irrespective of those fluctuations. Utilization measured over a single year may therefore provide an incomplete characterization of the activity or future value of a mature biospecimen repository.

This variability also argues against using a single utilization threshold as the basis for retention decisions. Previous studies have identified underutilization as an important challenge for biobanks (10–14), but utilization depends on the unit of measurement, observation period, collection type, and scientific purpose for which specimens were retained. A collection with little activity during one period may become highly utilized following the emergence of a new scientific question, technology, biomarker, or therapeutic target; conversely, collections accumulated for a once-active research program may become less relevant over time. ACC does not resolve these questions or assign scientific value to individual specimens or collections. Instead, it adds a quantitative infrastructure dimension to the information available when institutions evaluate long-term inventories.

This framework may support more systematic approaches to collection review and lifecycle management. Rather than considering storage expansion primarily as a response to diminishing physical capacity, repositories could periodically evaluate existing collections across multiple dimensions, including carrying cost, utilization history, uniqueness, replacement feasibility, consent and regulatory requirements, scientific relevance, quality, associated data, anticipated future demand, and strategic institutional importance [25]. Collections with low recent utilization but high scientific or replacement value may appropriately remain in long-term storage. In other circumstances, prolonged low utilization combined with limited scientific relevance, poor associated data, or readily replaceable material may prompt further review, consolidation, transfer, or responsible disposition as part of formal collection lifecycle management, where ethically and legally appropriate. In this context, ACC contributes the infrastructure and financial dimension of a multidimensional assessment rather than serving as a retention threshold or decision rule.

ACC may also inform storage technology and capital planning. Because the framework incorporates annualized equipment investment together with recurring operating requirements, repositories can evaluate how changes in equipment, storage modality, capacity, monitoring, or energy and cryogen requirements affect the resources necessary to sustain an inventory over time. This may become increasingly relevant as institutions confront aging storage equipment, competing demands for laboratory space, energy-use and sustainability goals, and pressure to use research infrastructure efficiently. The objective, however, is not necessarily to minimize ACC. Investments that increase resilience, specimen protection, traceability, or operational readiness may appropriately increase carrying cost. Making these investments visible instead allows institutions to understand the resources committed to long-term preservation and the operational functions those resources support.

Because ACC is based on locally incurred resource requirements rather than fixed monetary assumptions, the framework is intended to be adaptable across repositories operating in different geographic, economic, and regulatory environments. Absolute values will necessarily vary according to local currencies, storage technologies, energy and cryogen markets, infrastructure requirements, and institutional practices. The transferable element of ACC is therefore not a particular monetary benchmark, but a standardized approach to identifying and normalizing the recurring direct investments required to sustain long-term biospecimen storage. Application of the framework across institutions may consequently be most informative for understanding cost structure, capacity, and change over time rather than for direct comparison of absolute ACC values between repositories.

Several limitations should be considered when interpreting these findings. First, the analysis represents a single academic biobank, and absolute costs reflect institution-specific equipment, energy, cryogen, monitoring, maintenance, and inventory-management expenses. The reported ACC and normalized values therefore should not be interpreted as universal cost benchmarks. Second, ACC was intentionally restricted to direct infrastructure and operating expenses attributable to long-term storage and excluded personnel, facility and space costs, institutional overhead, facility-level emergency infrastructure, and costs associated with specimen acquisition, processing, and initial accessioning. ACC therefore does not represent the comprehensive institutional cost of operating a biobank; rather, it isolates the recurring direct investment associated with maintaining long-term storage infrastructure and stored inventory. Third, equipment acquisition costs were annualized using expected useful life, and actual equipment longevity may differ from these assumptions. Similarly, some recurring expenses were shared across storage infrastructure and required attribution to the evaluated repository. Equipment-specific operating costs may also vary according to storage technology, equipment age, configuration, occupancy, and operational role. For example, energy consumption may differ between fully and partially occupied mechanically refrigerated units, while contingency or reserve equipment may have different operating characteristics from routinely inventory-bearing systems. These factors may influence institution-specific ACC estimates and further support the use of locally measured operating costs when available.

The utilization analysis also has limitations. Retrieval activity was examined over a three-year period and may be strongly influenced by individual studies or large collection-level distributions. Vial-equivalent retrievals provide a standardized measure of material leaving storage but do not capture the scientific importance of individual specimens, the number or quality of resulting publications, subsequent discoveries, downstream clinical impact, or the future value of specimens that have not yet been retrieved. For box-level inventory records, each 9 × 9 inventory box was standardized to its nominal capacity of 81 vial-equivalent positions. Individual position-level occupancy within these boxes was not independently verified; therefore, the 81-position conversion represents an operational normalization for estimating occupied storage capacity rather than confirmation that every position within each box contained a vial or a measure of biological or scientific equivalence. Longer observation periods and application of ACC across repositories with different organizational structures, storage technologies, collection types, and regional cost environments will be important for assessing the broader utility and generalizability of the framework.

## 5. Conclusions

Long-term biospecimen preservation creates a recurring institutional obligation that persists beyond specimen acquisition and is not directly determined by annual utilization. Annual Carrying Cost (ACC) provides a practical framework for quantifying the direct infrastructure and operating costs associated with maintaining existing biospecimen inventories. When considered alongside storage capacity and retrieval activity, ACC can help institutions better characterize the resources committed to long-term specimen retention and distinguish necessary investments in resilience and reserve capacity from routinely occupied storage.

ACC is not intended to assign monetary value to biospecimens or to determine whether individual specimens or collections should be retained. Rather, it provides an additional operational measure that can be considered alongside scientific value, uniqueness, utilization, replacement feasibility, and anticipated future demand. Incorporating carrying cost into broader collection and infrastructure assessments may support more informed capacity planning, lifecycle management, resource allocation, and sustainable long-term biospecimen stewardship.

## Figures and Tables

**Figure 1:**
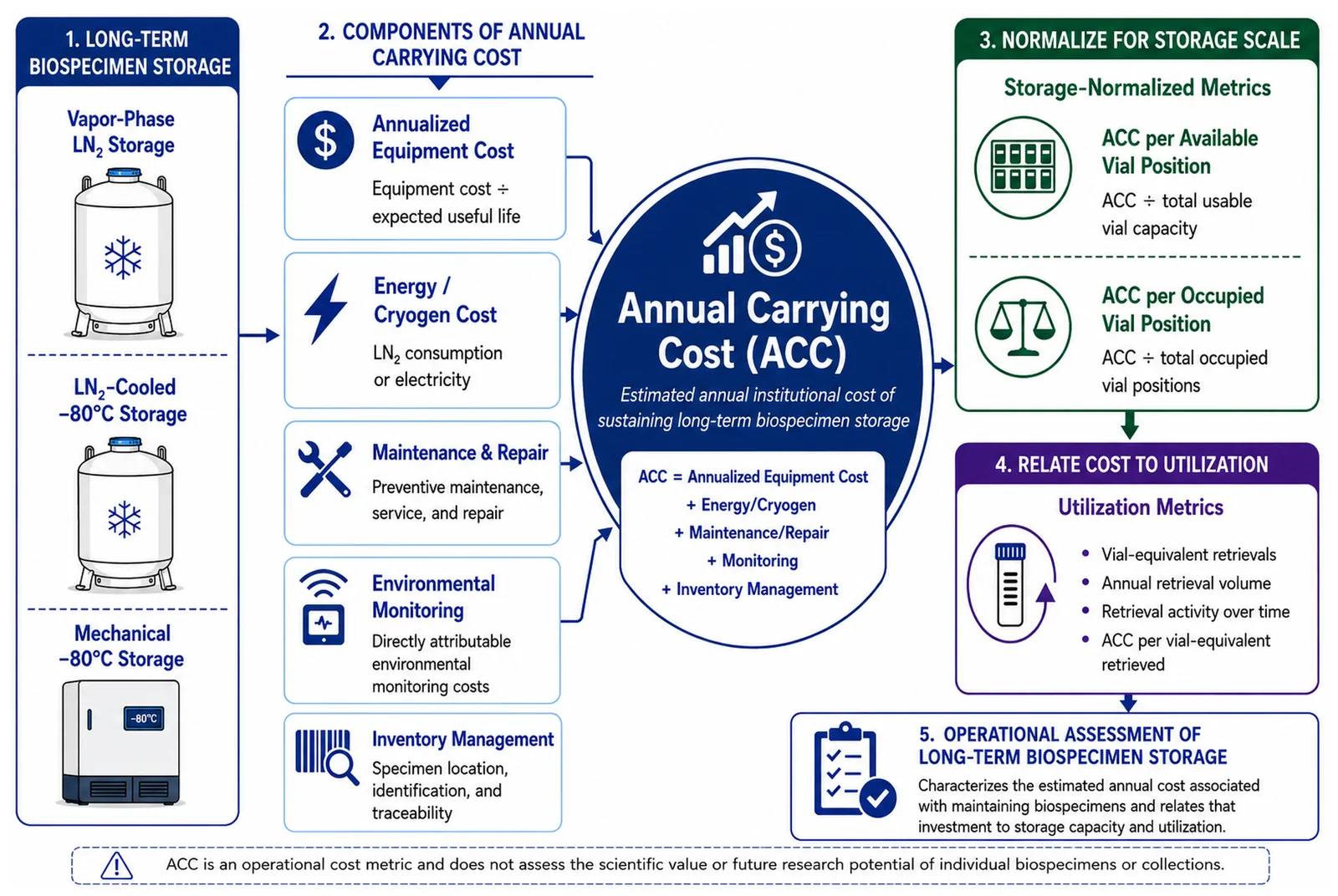
**Conceptual framework for Annual Carrying Cost (ACC) of long-term biospecimen storage.** ACC integrates recurring direct infrastructure and operating costs associated with long-term biospecimen preservation and relates this investment to maintained storage capacity, occupied inventory, and research-directed utilization. The framework is applicable across storage technologies and is intended as an operational cost metric rather than a measure of the scientific value or future research potential of individual biospecimens or collections.

**Figure 2:**
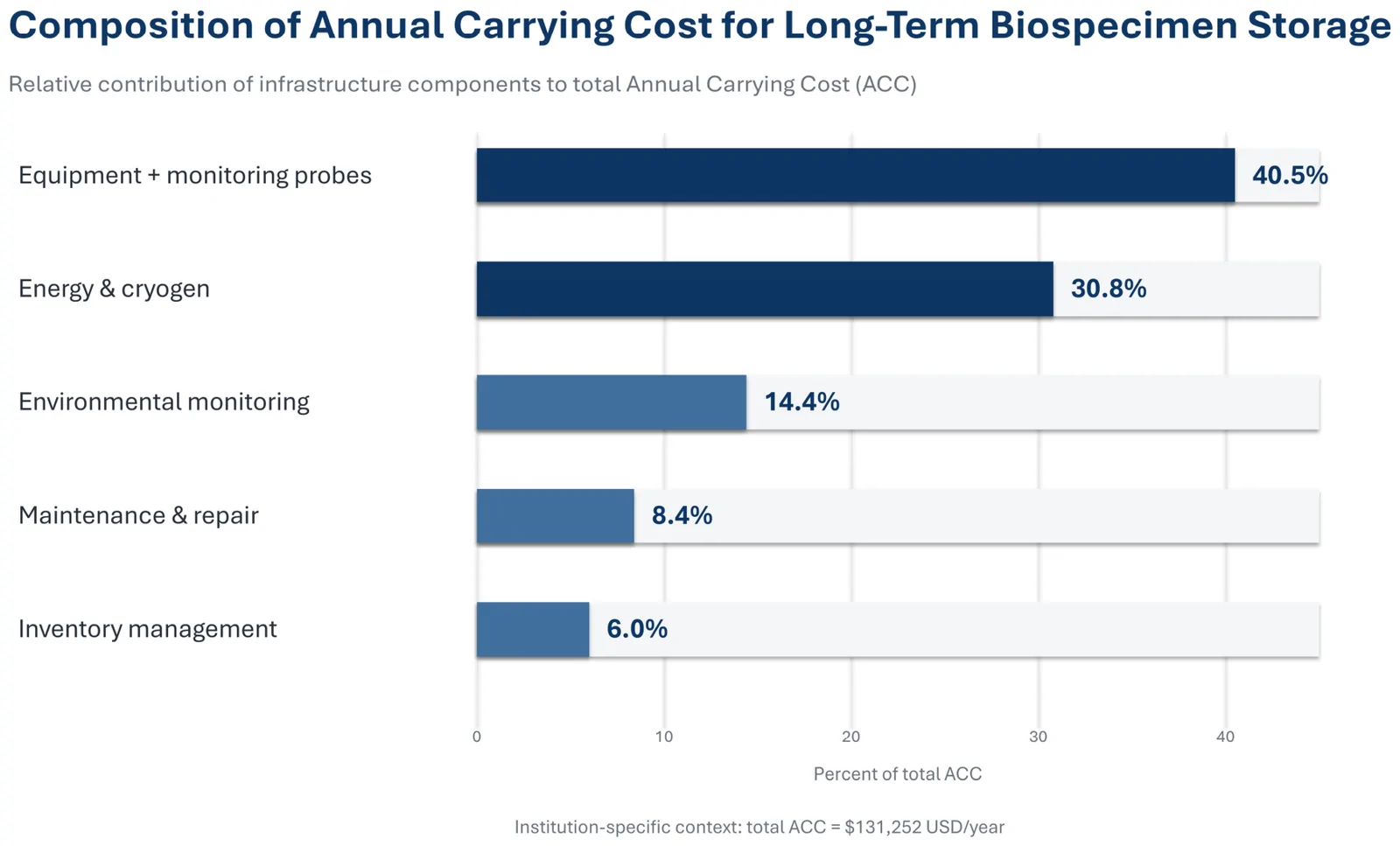
**Relative composition of Annual Carrying Cost for long-term biospecimen storage.** Annualized equipment costs, including dedicated environmental monitoring probes, represented 40.5% of total Annual Carrying Cost (ACC), followed by energy and cryogen (30.8%), environmental monitoring (14.4%), maintenance and repair (8.4%), and inventory management (6.0%). Total ACC for the evaluated storage infrastructure was $131,252 USD annually. Percentages represent the relative contribution of each infrastructure component to total ACC; absolute costs reflect institution-specific infrastructure and pricing.

**Figure 3:**
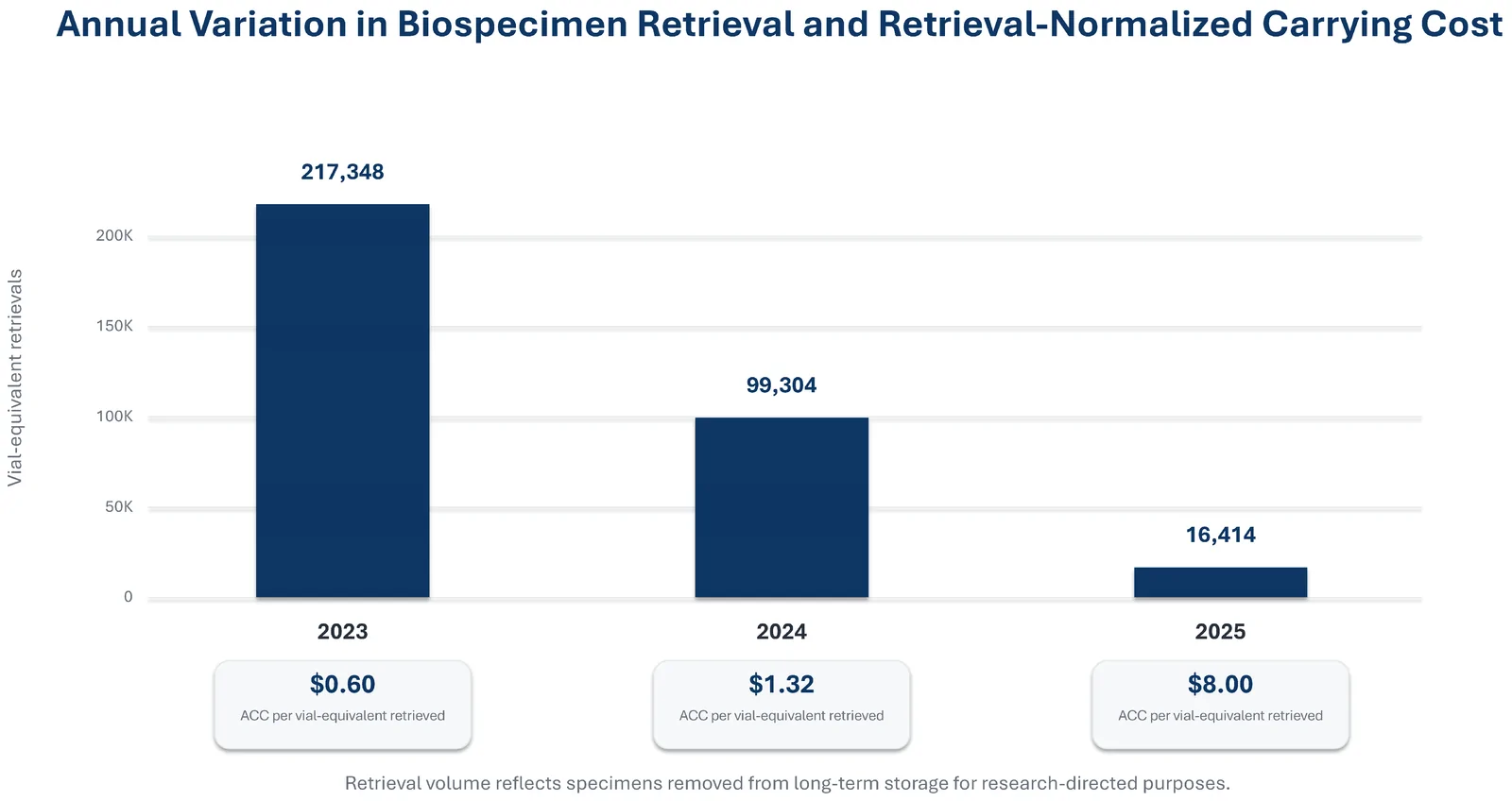
**Annual biospecimen retrieval volume and retrieval-normalized Annual Carrying Cost.** Vial-equivalent retrieval volume varied substantially across the three-year study period, from 217,348 vial-equivalent retrievals in 2023 to 99,304 in 2024 and 16,414 in 2025. Annual Carrying Cost (ACC) remained $131,252, resulting in retrieval-normalized ACC values of $0.60, $1.32, and $8.00 per vial-equivalent retrieved in 2023, 2024, and 2025, respectively. Vial-equivalent retrievals represent biospecimens removed from long-term storage for research-directed purposes, with retrieved inventory boxes standardized to 81 vial equivalents. Variation in retrieval-normalized ACC reflects annual differences in specimen utilization rather than changes in the underlying storage infrastructure.

**Table 1:** **Operational Function and Capacity of Long-Term Biospecimen Storage Infrastructure.** Storage infrastructure was classified according to operational function at the time of inventory assessment. Inventory-bearing units were maintained at operating conditions and contained routine biospecimen inventory. Online contingency units were maintained at operating temperature without routine inventory to provide immediately available capacity for emergency specimen transfer and short-term fluctuations in storage demand. Offline/staged reserve units contained no routine inventory and were not maintained at operating conditions but remained installed and available for activation in response to future storage requirements. Occupied vial positions include individually inventoried vials and inventory boxes standardized to 81 vial-equivalent positions. Occupancy was calculated as occupied vial positions divided by usable capacity within each operational category. LN_2_, liquid nitrogen; ULT, ultra-low temperature.

Operational Function	Storage Status	Units, n	Usable Capacity, Vial Positions	Occupied Vial Positions	Occupancy, %
Inventory-bearing	Maintained at operating conditions; routine biospecimen inventory	8	485,720	272,476	56.1%
Online contingency	Maintained at operating temperature; intentionally no routine inventory		116,800	0	0.0%
Offline/staged reserve	Installed and available for activation; not maintained at operating conditions		229,600	0	0.0%
Total storage infrastructure	All inventory-bearing, contingency, and staged reserve capacity	16	832,120	272,476	32.7%

*Occupied vial positions include individually inventoried vials and inventory boxes standardized to 81 vial-equivalent positions.* Inventory-bearing units contained routine biospecimen inventory. Online contingency units were maintained at operating temperature without routine inventory to provide immediately available emergency and short-term capacity. Offline/staged reserve units contained no inventory and were not maintained at operating conditions but remained installed and available for future activation. Overall physical capacity utilization was calculated as occupied vial positions divided by total available capacity. Occupancy of inventory-bearing storage was calculated separately to distinguish actively populated storage from capacity intentionally maintained for contingency and future requirements. Across the repository, 346,400 vial positions (41.6% of total capacity) were intentionally maintained as online contingency or offline/staged reserve infrastructure.

## Data Availability

The original contributions presented in this study are included in the article. Further inquiries can be directed to the corresponding author.
